# Development and validation of machine learning models to predict 30-day mortality in patients with cardiac arrest complicated by acute kidney injury

**DOI:** 10.1016/j.clinsp.2026.101129

**Published:** 2026-09-08

**Authors:** Meng Yuan, Lei Zhong, Jie Min, Mingxia Ni, Yufeng Yao

**Affiliations:** a Department of Intensive Care Unit, Huzhou Central Hospital, Affiliated Central Hospital of Huzhou University, Zhejiang, China; b Department of Operating Room, Huzhou Central Hospital, Affiliated Central Hospital of Huzhou University, Zhejiang, China; c Department of Anesthesiology, Huzhou Central Hospital, Affiliated Central Hospital of Huzhou University, Zhejiang, China; d Fifth School of Clinical Medicine of Zhejiang Chinese Medical University, Zhejiang, China

**Keywords:** Cardiac arrest, Acute kidney injury, Machine learning, Mortality, Predictive model

## Abstract

**Background:**

Cardiac Arrest (CA) often leads to Acute Kidney Injury (AKI), presenting a major health issue. This study aims to develop and validate machine learning models predicting 30-day mortality in CA patients with AKI.

**Methods:**

A retrospective study was conducted on 1,121 adult ICU patients diagnosed with CA and AKI using data from the MIMIC-IV database. Data from 2008‒2016 (n = 900) formed the training cohort, while 2017‒2019 data (n = 221) served as the temporal validation cohort. Feature selection utilized LASSO regression, followed by the application of six machine learning algorithms. Model performance was evaluated using ROC curves, calibration curves, and decision curve analysis, with comparisons made against the APACHE II and SOFA scores.

**Results:**

Seven key features were identified, namely, APACHE II score, lactate, anion gap, RDW, oliguria, norepinephrine, and temporary pacemaker implantation. The LASSO-Logistic Regression (LASSO-LR) model demonstrated the most stable performance in validation (AUROC = 0.751), significantly outperforming APACHE II (0.583, p < 0.001) and SOFA (0.660, p = 0.009), with a Brier score of 0.209. SHAP analysis revealed oliguria and RDW as the most significant predictors.

**Conclusions:**

The LASSO-LR model offers a modest but statistically significant improvement over conventional scoring systems for predicting 30-day mortality in patients with CA and AKI, with acceptable calibration and interpretability. However, the clinical meaningfulness of this incremental benefit requires prospective validation before clinical implementation.

## Highlights

• LASSO-LR predicts 30-day mortality in CA-AKI (AUROC = 0.751, Brier = 0.209).
• LASSO-LR outperforms APACHE II (0.583) and SOFA (0.660).
• SHAP and nomogram reveal oliguria and RDW as top predictors.

## Introduction

Cardiac Arrest (CA) represents a critical, life-threatening global public health concern, often culminating in elevated mortality rates and significant long-term morbidities.^1^ Global statistics indicate that approximately 4.5-million individuals succumb to CA annually, with an estimated 544,000 cases reported in China.^2^ Despite notable advancements in Cardiopulmonary Resuscitation (CPR) techniques and subsequent therapeutic interventions in recent years, the prognosis remains dismal. Merely about 20% of patients experiencing Out-of-Hospital Cardiac Arrest (OHCA) achieve Return of Spontaneous Circulation (ROSC), and of those, only 10% survive to hospital discharge.^3^ Acute Kidney Injury (AKI), a prevalent and severe complication in patients with CA, is significantly correlated with the progression of chronic kidney disease and adverse prognostic outcomes.^4^^,^^5^ Empirical evidence indicates that the incidence of AKI among OHCA patients varies between 33% and 48%, with its presence being strongly associated with elevated 30-day and 1-year mortality rates.^5^^,^^6^ Consequently, evaluating mortality risk in CA patients who concurrently experience AKI is critically important in clinical practice.

Currently, despite the availability of prognostic scoring systems for patients experiencing CA, including the OHCA score,^7^ the Cardiac Arrest Hospital Prognosis (CAHP) score,^8^ the Acute Physiology and Chronic Health Evaluation II (APACHE II) score,^9^ and the Sequential Organ Failure Assessment (SOFA) score,^10^ these tools exhibit limitations in managing complex, multidimensional clinical data, particularly concerning personalized prediction and non-linear data processing.^11^^,^^12^ With the advancement of computational technology and data science, the implementation of machine learning techniques in the medical field has become increasingly prevalent, demonstrating significant potential in disease risk prediction. Recent studies have predominantly concentrated on risk prediction models for CA.^13^^,^^14^ Notably, four predictors have been identified for the development of AKI following CA: Chronic Kidney Disease (CKD), albumin level, shock, and heart rate.^15^ However, once AKI is established, the determinants of mortality may change. While certain indicators, such as shock, likely continue to play a crucial role, mortality in these patients is probably influenced by more complex pathophysiological processes, including multi-organ dysfunction and acute physiological derangements. Despite this understanding, there is a notable absence of machine learning-based prediction models specifically designed to predict 30-day mortality in patients with CA complicated by AKI.

This study aims to develop and validate a predictive model based on machine learning algorithms to enhance the accuracy of predicting the 30-day mortality risk for patients with CA complicated by AKI. By comprehensively analyzing clinical data, this study will provide clinicians with an additional tool to better assess patient prognosis, thereby offering a reference for the individualized management of CA patients with AKI.

## Methods

### Data source

This retrospective single-center clinical investigation was conducted in accordance with the Transparent Reporting of a Multivariable Prediction Model for Individual Prognosis or Diagnosis (TRIPOD) statement^16^ for model development and validation, and the Standards for Reporting Diagnostic Accuracy (STARD) guidelines for prognostic studies. Data were sourced from the Medical Information Mart for Intensive Care IV version 2.0 (MIMIC-IV 2.0),^17^ a database established by the Massachusetts Institute of Technology (MIT) Laboratory for Computational Physiology. This database includes anonymized data from 76,943 ICU admissions at the Beth Israel Deaconess Medical Center (BIDMC) in Boston, Massachusetts, collected between 2008 and 2019. To protect patient privacy, all identifiable information was removed in compliance with established regulations. The use of this database was approved by the Institutional Review Boards (IRB) of BIDMC and MIT. The authors completed compliance training and were certified through the Collaborative Institutional Training Initiative (CITI) program to ensure adherence to ethical standards in human research and gained access to the database (certification ID: 53446653, 51774135, 51168595).

### Study population

**Fig. 1:**
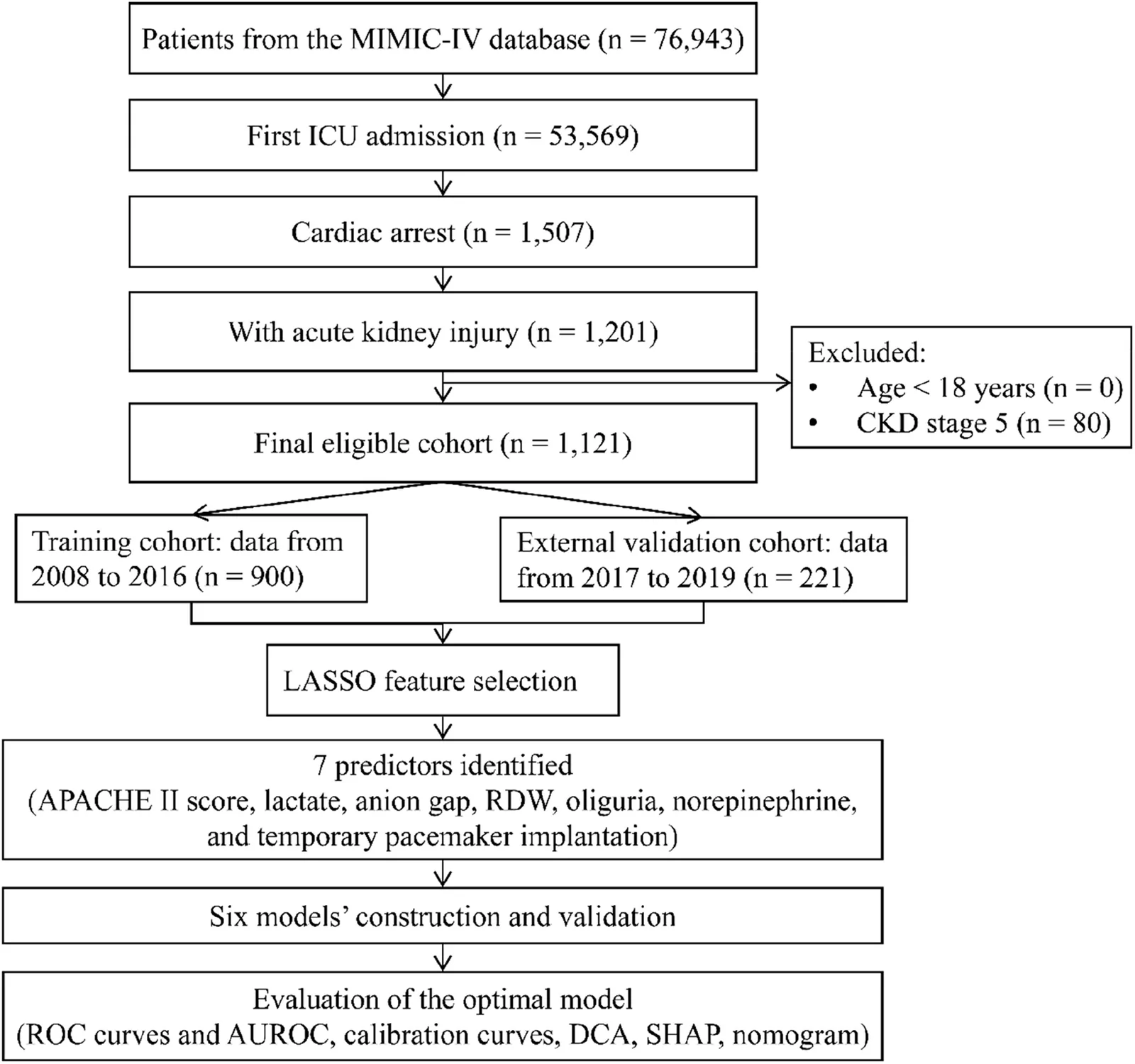
Overview of patient selection and model validation processes. MIMIC-IV, Medical Information Mart for Intensive Care IV; ICU, Intensive Care Unit; CKD, Chronic Kidney Disease, LASSO, Least Absolute Shrinkage and Selection Operator; APACHE II, Acute Physiology and Chronic Health Evaluation-II, RDW, Red Cell Distribution Width; ROC; Receiver Operating Characteristic; AUROC, Area Under the Receiver Operating Characteristic Curve; DCA, Decision Curve Analysis; SHAP, SHapley Additive exPlanations.

The study included 1,121 critically ill patients (aged ≥ 18-years) with CA complicated by AKI from the Intensive Care Unit (ICU). Patients were excluded if they had end-stage CKD (CKD stage 5, n = 80). Following a chronological order, patients from 2008 to 2016 (n = 900) were used to construct the training cohort, while those from 2017 to 2019 (n = 221) served as the temporal validation cohort to evaluate the model's performance. Fig. 1 details the patient selection process and the flowchart for model construction and validation.

### Diagnosis and outcomes

According to the Kidney Disease Improving Global Outcomes (KDIGO) criteria,^18^ AKI is defined by any of the following conditions: 1) An increase in serum creatinine by ≥ 0.3 mg/dL (≥ 26.5 μmoL/L) within 48-hours; 2) An increase in serum creatinine to ≥ 1.5 times the baseline level within the past 7-days; or 3) Urine output ≤ 0.5 mL/kg/h for 6-hours. Baseline creatinine values were sourced from the official MIMIC-IV database. The KDIGO criteria were specifically employed to delineate the study population. Additionally, oliguria, defined as a urine output of less than 400 milliliters in 24-hours, was considered as a potential predictor based on clinical practice and previous literature.^19^

The primary endpoint of this study was 30-day mortality. This time frame was chosen for several reasons. Firstly, it aligns with the Core Outcome Set for Cardiac Arrest (COSCA) consensus recommendation, which designates survival at 30-days or hospital discharge as a core outcome for evaluating the effectiveness of cardiac arrest interventions.^20^ Secondly, 30-day mortality is a widely utilized measure in contemporary cardiac arrest research, thereby facilitating comparisons across studies.^21^, ^22^, ^23^ Thirdly, this endpoint captures both early hemodynamic compromise and neurological injury while minimizing confounding factors associated with long-term comorbidities.

### Variable extraction

In this study, PostgreSQL software (version 14.5) was used to retrieve and process data from the MIMIC-IV database. For variables with a missing rate of less than 25%, multiple imputation was performed using the predictive mean matching method via the 'mice' package in R. Five imputed datasets (m = 5) were generated using 'mice' package, with 50 iterations conducted to ensure convergence. Least Absolute Shrinkage and Selection Operator (LASSO) regression was applied separately to each dataset for feature selection, and the same seven predictors were selected in all five datasets (selection frequency 100%). Multivariable logistic regression was then fitted to each dataset, and the results were pooled using Rubin’s rules to obtain final coefficient estimates. Since the results were highly consistent across datasets, the fourth imputed dataset was chosen as the representative dataset because its regression coefficients were closest to the pooled estimates. All subsequent analyses, including model training, hyperparameter tuning, performance evaluation, and SHAP explanation, were conducted on this representative dataset. Comprehensive statistics on the missing data are presented in Supplementary Table 1, while a comparison of the datasets before and after imputation is illustrated in Supplementary Fig. 1.

The predictive model was constructed using clinical and laboratory data collected within the first 24-hours of ICU admission. The dataset encompassed the following categories of variables: 1) Demographic data (age, sex, marital status); 2) Clinical scores (SOFA and APACHE II); 3) Vital signs (heart rate and mean arterial pressure); 4) Laboratory parameters, including arterial blood gas, complete blood count, renal function, liver function, coagulation profile, electrolytes, and glucose levels; 5) Comorbidities (e.g., diabetes, hypertension, cardiogenic shock, atrial fibrillation, etc.); and 6) Therapeutic interventions (e.g., mechanical ventilation, norepinephrine, temporary pacemaker implantation, etc.). Baseline creatinine values and AKI stages were recorded, oliguria was defined based on urine criteria, and 30-day mortality was assessed as the outcome measure. Comorbidities were identified using International Classification of Diseases, Ninth and Tenth Revision (ICD-9/10) codes.

### Statistical analysis

Quantitative data, characterized by their distribution, were analyzed accordingly: parameters with a normal distribution were detailed as mean ± standard deviation and subjected to independent samples *t*-tests, while those not normally distributed were depicted as median and interquartile range, analyzed using Mann-Whitney *U*-tests. Categorical variables were tabulated as frequency and percentage, with analysis performed using the Chi-Square test or Fisher's exact test, where appropriate.

To circumvent the risk of overfitting, feature selection was meticulously conducted employing LASSO regression with cross-validation. The Events-Per-Variable (EPV) ratio in the training cohort was approximately 71.7 (502-events for 7-predictors), well above the recommended minimum threshold, indicating adequate statistical power for model development. To ensure reproducibility of the feature selection process, a fixed random seed (set.seed(123)) was used during the cross-validation procedure. Hyperparameter tuning was systematically executed using the grid search method in combination with five-fold cross-validation, culminating in the identification of the optimal hyperparameter configuration. Six predictive models were subsequently developed: Decision Tree (DT), extreme gradient boosting (XGBoost), Support Vector Machine (SVM), light gradient boosting machine (LightGBM), K-Nearest Neighbor (KNN), and LASSO-Logistic Regression (LASSO-LR). Model performance was assessed in terms of discrimination, calibration, and clinical utility. Discrimination was quantified using the Receiver Operating Characteristic (ROC) curve and the Area Under the ROC Curve (AUROC). Calibration was evaluated via calibration plots and Brier score. Decision Curve Analysis (DCA) was employed to assess the clinical net benefit of each model across a range of threshold probabilities.

To benchmark our machine learning models against current clinical practice, we additionally evaluated the predictive performance of the APACHE II and SOFA scores. For each score, the total value was entered as a single predictor in a univariate logistic regression model fitted in the training cohort; the resulting predicted probabilities were then applied to the temporal validation cohort. Discriminatory ability was measured by AUROC and compared with that of the optimal machine learning model (LASSO-LR) using DeLong's test. Furthermore, continuous Net Reclassification Improvement (NRI) and Integrated Discrimination Improvement (IDI) were computed to assess the incremental clinical value of the LASSO-LR model beyond these traditional scores. Calibration and clinical utility of these scores were also examined using calibration curves and DCA.

After identifying LASSO-LR as the optimal model based on the above comparisons, we further enhanced its interpretability. SHapley Additive exPlanations (SHAP) were applied to the LASSO-LR model to visualize the impact of individual features on predictions. Additionally, a nomogram was constructed based on the LASSO-LR model to provide a clinically accessible tool for estimating 30-day mortality risk.

Data manipulation and all statistical analysis were conducted utilizing R software (version 4.2.3), with statistical significance demarcated at p < 0.05.

## Results

### Patient characteristics

This study included 1,121 critically ill patients with CA complicated by AKI, with an average age of 66.98±16.91 years, and 60.39% (677) were male. The 30-day mortality rate was 54.68% (n = 613). Patients were divided into a training cohort (n = 900) and a temporal validation cohort (n = 221) in chronological order. Table 1 presents the baseline characteristics for selected key variables, while the complete list of all candidate variables is provided in Supplementary Table 2.

**Table 1:** The baseline characteristics for selected key variables.

**Characteristic**	**Total (n = 1,121)**	**Training cohort (n = 900)**	**Temporal validation cohort (n = 221)**	**t/Z/χ^2^**	**p-value**
Age (years)	66.98 ± 16.91	67.76 ± 16.68	63.82 ± 17.50	3.113	0.002
Male, n (%)	677 (60.39)	542 (60.22)	135 (61.09)	0.055	0.814
SOFA score	9.20 ± 4.39	9.14 ± 4.43	9.42 ± 4.20	-0.843	0.400
APACHE II score	26.91 ± 8.34	27.20 ± 8.55	25.74 ± 7.30	2.331	0.020
30-day mortality (%)	613 (54.68)	502 (55.78)	111 (50.23)	2.207	0.137
Baseline creatinine (umol/L)	71.99 (53.04, 95.83)	73.67 (39.78, 96.98)	70.72 (35.36, 93.02)	1.524	0.127
AKI stages, n (%)				6.738	0.034
Stage 1	187 (16.68)	141 (15.67)	46 (20.81)		
Stage 2	485 (43.26)	383 (42.56)	102 (46.15)		
Stage 3	449 (40.05)	376 (41.78)	73 (33.03)		
Lactate (mmoL/L)	3.00 (1.70, 5.80)	2.90 (1.20, 5.55)	3.70 (1.40, 6.30)	-3.012	0.003
Anion gap (mmoL/L)	17.95 ± 5.67	17.94 ± 5.66	18.00 ± 5.73	-0.130	0.896
RDW (%)	15.00 ± 2.26	15.05 ± 2.24	14.81 ± 2.32	1.377	0.169
Oliguria, n (%)	207 (18.47)	176 (19.56)	31 (14.03)	3.602	0.058
Temporary pacemaker implantation, n (%)	42 (3.75)	33 (3.67)	9 (4.07)	0.081	0.776
Norepinephrine, n (%)	700 (62.44)	542 (60.22)	158 (71.49)	9.611	0.002

SOFA, Sequential Organ Failure Assessment; APACHE II, Acute Physiology and Chronic Health Evaluation-II; AKI, Acute Kidney Injury; RDW, Red cell Distribution Width.

The 30-day mortality rates for the training and temporal validation cohorts were 55.78% and 50.23%, respectively, showing no statistically significant difference (*p* = 0.137). Among the variables ultimately selected for model development, significant differences between the two cohorts were observed in APACHE II score, norepinephrine, and lactate levels (*p* < 0.05), while anion gap, Red cell Distribution Width (RDW), oliguria, and temporary pacemaker implantation were comparable between groups (*p* > 0.05).

### Feature selection

The glmnet package in R was used to perform LASSO regression with cross-validation analysis on the 30-day mortality of ICU patients with CA complicated by AKI. In the study, 30-day mortality was used as the dependent variable, and 56 features from Supplementary Table 2 were used as independent variables. Cross-validation determined the optimal lambda (λ) value to be 0.058 (Lambda.1se, as shown in Supplementary Fig. 2). Ultimately, seven key features were selected: APACHE II score, blood lactate, anion gap, RDW, oliguria, norepinephrine, and temporary pacemaker implantation.

### Sensitivity analysis for APACHE II exclusion

To assess whether the inclusion of APACHE II biased the feature selection, we repeated the LASSO procedure after excluding it from the candidate variables. The selected features were lactate, anion gap, BUN, RDW, oliguria, norepinephrine, and temporary pacemaker ‒ largely overlapping with the seven predictors identified in the original LASSO selection. In the training cohort, this sensitivity model achieved an AUROC of 0.736 (95% CI: 0.703‒0.768); in the validation cohort, it achieved an AUROC of 0.769 (95% CI: 0.708‒0.831).

### Models’ construction and validation

The seven key features selected in this study were applied to six machine learning algorithms for training: DT, XGBoost, SVM, LightGBM, KNN, and LASSO-LR. Each model was trained using the training cohort and subsequently assessed on the temporal validation cohort using five-fold cross-validation. Table 2 provides a summary of the performance metrics for all evaluated models, encompassing the six machine learning models as well as the two clinical scoring systems, APACHE II and SOFA.

**Table 2:** Performance comparison of predictive models for 30-day mortality in the training cohort and the temporal validation cohort.

**Models**	**AUROC (95%CI)**	**Sensitivity**	**Specificity**	**Accuracy**	**Kappa**
Training cohort					
Decision tree	0.828 (0.802‒0.854)	0.727	0.744	0.734	0.467
XGBoost	0.742 (0.710‒0.774)	0.745	0.613	0.687	0.361
Support vector machine	0.737 (0.705‒0.770)	0.757	0.608	0.691	0.368
LightGBM	0.756 (0.725‒0.787)	0.767	0.616	0.700	0.386
K-nearest neighbor	0.889 (0.869‒0.909)	0.803	0.804	0.803	0.604
Lasso-LR	0.742 (0.710‒0.774)	0.805	0.540	0.688	0.353
APACHE II sore	0.651 (0.615‒0.687)	0.749	0.490	0.634	0.244
SOFA sore	0.623 (0.587‒0.660)	0.558	0.631	0.590	0.185
Temporal validation cohort					
Decision tree	0.663 (0.592‒0.734)	0.532	0.682	0.606	0.213
XGBoost	0.724 (0.657‒0.791)	0.739	0.627	0.683	0.366
Support vector machine	0.723 (0.656‒0.790)	0.757	0.582	0.670	0.339
LightGBM	0.731 (0.665‒0.797)	0.730	0.618	0.674	0.348
K-nearest neighbor	0.667 (0.596‒0.738)	0.631	0.618	0.624	0.249
LASSO-LR	0.751 (0.687‒0.816)	0.838	0.509	0.674	0.347
APACHE II sore	0.583 (0.508‒0.658)	0.586	0.573	0.579	0.158
SOFA sore	0.660 (0.589‒0.731)	0.802	0.445	0.624	0.248

AUROC, Area Under the Receiver Operating Characteristic Curve; CI, Confidence Interval; XGBoost, Extreme Gradient Boosting, LightGBM, Light Gradient Boosting Machine; LASSO-LR, Least Absolute Shrinkage and Selection Operator-Logistic Regression; APACHEII, Acute Physiology and Chronic Health Evaluation-II; SOFA, Sequential Organ Failure Assessment.

In the training cohort, all six machine learning models attained AUROCs exceeding 0.73. The KNN model exhibited the highest discriminatory power with an AUROC of 0.889 (95% Confidence Interval [95% CI: 0.869‒0.909]), while the SVM model demonstrated the lowest performance among them, with an AUROC of 0.737 (95% CI: 0.705‒0.770). Notably, several complex models displayed significant discrepancies between training and validation performances. Specifically, the KNN and DT models experienced substantial reductions in AUROC from training to validation, with decreases of 0.222 and 0.165, respectively, indicating pronounced overfitting. Conversely, the Lasso-LR model exhibited remarkable stability, maintaining nearly identical AUROCs in both training 0.742 (95% CI: 0.710‒0.774) and validation 0.751 (95% CI: 0.687‒0.816) phases. This stability highlights its resistance to overfitting, despite not achieving the highest raw performance in either cohort.

In the temporal validation cohort, the LASSO-LR model achieved the highest AUROC among all evaluated models (0.751). This was followed by the ensemble methods, with LightGBM and XGBoost achieving AUROCs of 0.731 and 0.724, respectively. In contrast, the DT model exhibited the lowest performance (AUROC = 0.663, 95% CI: 0.592‒0.734), as shown in Supplementary Fig. 3. Calibration analysis indicated that the Lasso-LR model was well-calibrated, as evidenced by a Brier score of 0.209 in the validation cohort (Supplementary Fig. 4). The Hosmer-Lemeshow goodness-of-fit test further confirmed adequate calibration, with p = 0.145 in the training cohort and p = 0.118 in the validation cohort. Furthermore, DCA revealed that the Lasso-LR model provided a positive net benefit across a range of threshold probabilities (Supplementary Fig. 5).

When compared to traditional clinical scoring systems, the LASSO-LR model showed statistically significant but modestly improved discrimination relative to the APACHE II score (AUROC = 0.583, 95% CI: 0.508‒0.658; DeLong's test *p* < 0.001) and the SOFA score (AUROC = 0.660, 95% CI: 0.589‒0.731; p = 0.009) in the validation cohort. Furthermore, continuous NRI and IDI analyses demonstrated significant incremental clinical value of the LASSO-LR model over APACHE II (continuous NRI = 0.624, 95% CI 0.374‒0.874; IDI = 0.197, 95% CI 0.144‒0.249; both *p* < 0.001) and SOFA (continuous NRI = 0.679, 95% CI 0.431‒0.927; IDI = 0.135, 95% CI 0.089‒0.181; both *p* < 0.001). Supplementary Fig.s 6‒11 present the ROC curves, calibration plots, and decision curves for these clinical scores in both the training and validation cohorts. Supplementary Table 3 provides detailed comparisons between the LASSO-LR model and all other models, including differences in AUROC with 95% CIs and results from DeLong's test.

### Sensitivity analysis for temporary pacemaker

To assess the influence of the temporary pacemaker variable on model performance, we performed two sensitivity analyses. First, excluding all patients with a temporary pacemaker (n = 42) yielded model performance comparable to the original model, with Lasso-LR achieving an AUROC of 0.730 (95% CI: 0.697‒0.764) and 0.738 (95% CI: 0.671‒0.806) in the validation cohort (Supplementary Table 4; Supplementary Figs. 14‒17). Second, recoding temporary pacemaker as “not present” for these patients while retaining them in the cohort also left model performance essentially unchanged: AUROC 0.734 (95% CI: 0.701‒0.766) in the training cohort and 0.741 (95% CI: 0.676‒0.807) in the validation cohort (Supplementary Table 5).

### SHAP interpretation in the optimal model

**Fig. 2:**
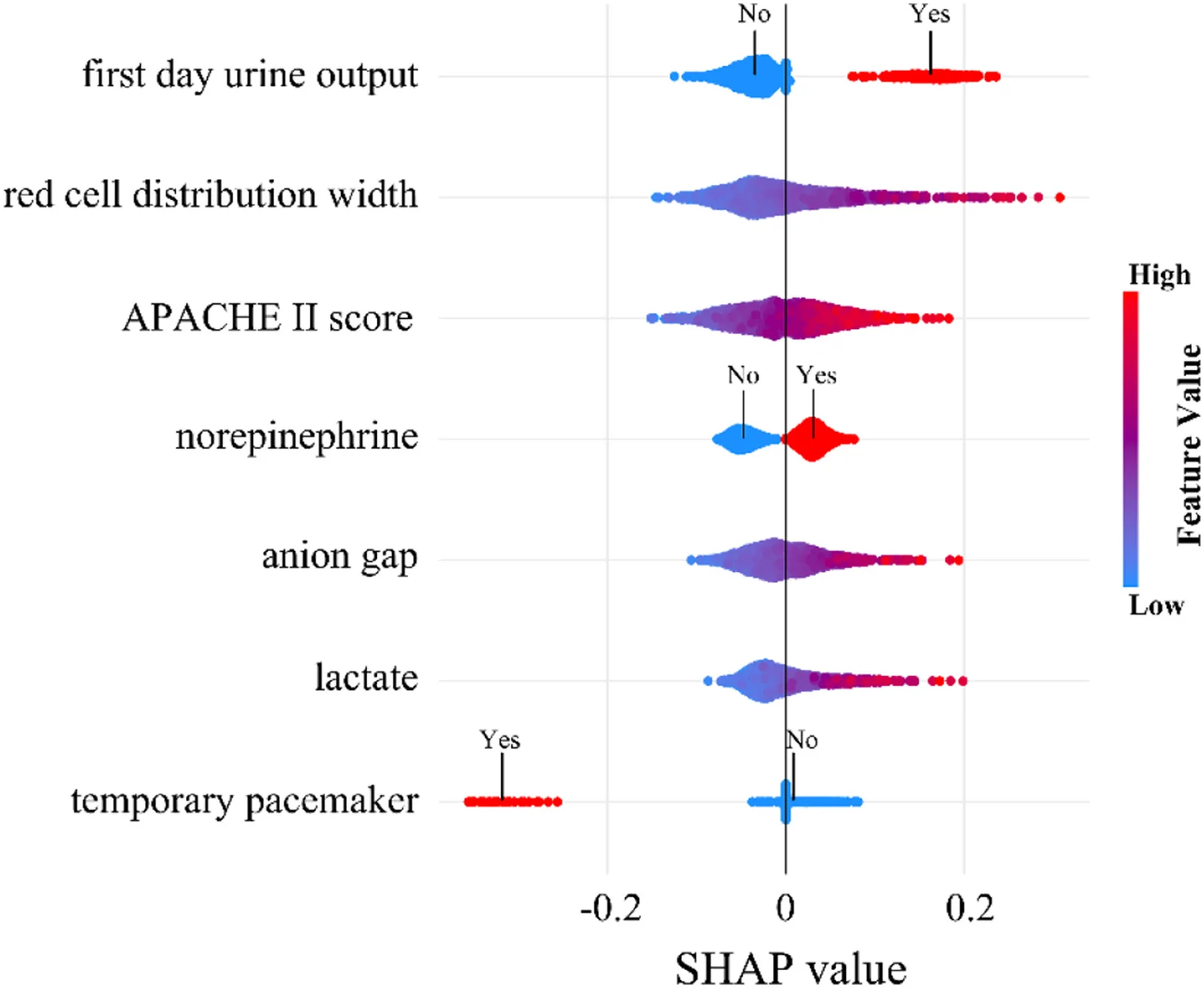
SHAP beeswarm plot of feature importance in the Lasso-LR model. Each dot represents an individual patient. The horizontal axis represents the SHAP value, indicating the impact of each feature on the model output (positive SHAP values correspond to increased mortality risk). Features are ordered on the vertical axis by their mean absolute SHAP values, reflecting their importance in the model. Color indicates the feature value for each patient: red represents higher values (or presence for categorical variables), and blue represents lower values (or absence for categorical variables). SHAP, SHapley Additive exPlanations; APACHE II, Acute Physiology and Chronic Health Evaluation-II.

Utilizing the SHAP software package for an in-depth analysis of the LASSO-LR model, the individual impact of each feature within the sample was quantified by SHAP values. Elevated SHAP values indicate an increased risk of mortality. The contribution of each predictive variable to the mortality rate can be interpreted as its cumulative effect on the overall risk. Fig. 2 illustrates a SHAP beeswarm plot that depicts the distribution of SHAP values for each feature. Features are ranked according to their mean absolute SHAP values, underscoring their significance in the model prediction: oliguria, RDW, APACHE II score, norepinephrine, anion gap, lactate, and temporary pacemaker implantation. Supplementary Fig. 12 further elucidates the influence of these features on the predicted outcomes. Notably, temporary pacemaker implantation is associated with a lower SHAP value, suggesting a negative correlation with increased 30-day mortality risk in patients with CA complicated by AKI. However, this association is likely indicative of patient selection bias rather than a causal protective effect. In contrast, the remaining six features exhibit a positive correlation with higher SHAP values, indicating that the augmentation of these factors is associated with an elevated 30-day mortality risk.

### Nomogram construction and evaluation

**Fig. 3:**
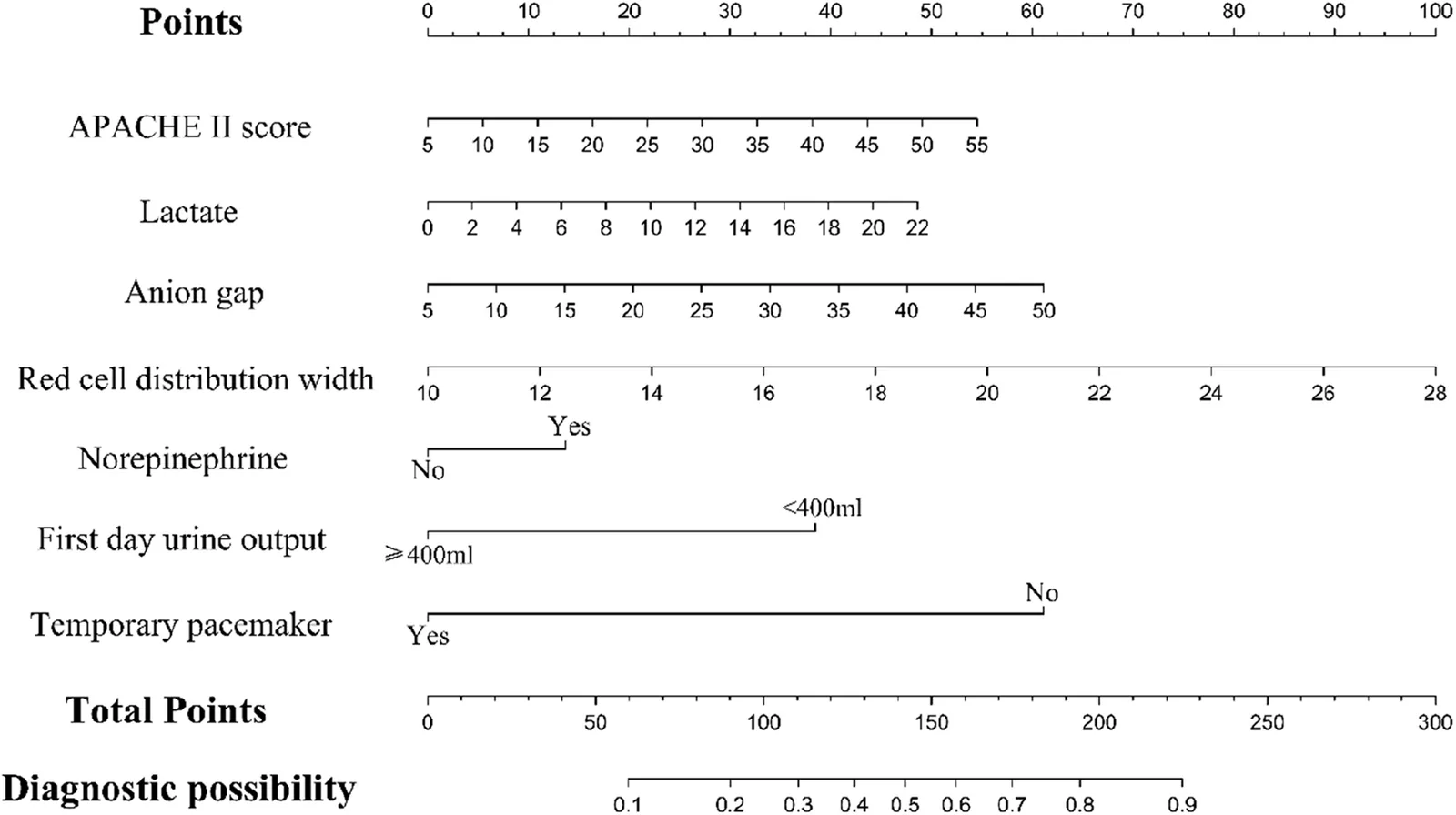
Nomogram for predicting the 30-day mortality risk of death in patients with cardiac arrest complicated by acute kidney injury. APACHE II, Acute Physiology and Chronic Health Evaluation-II.

**Fig. 4:**
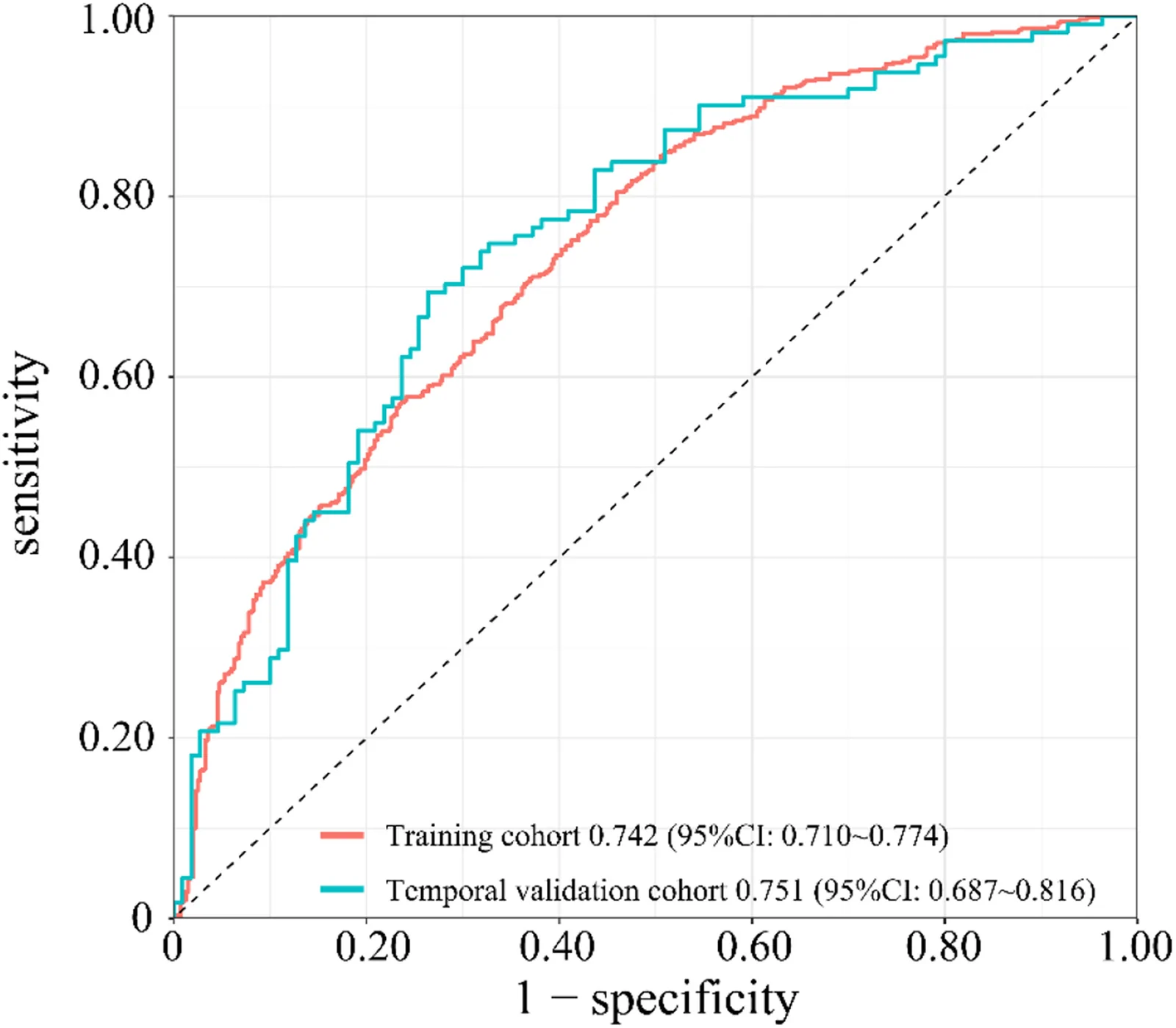
ROC curves and AUROCs of the Lasso-LR model in the training cohort and temporal validation cohort. ROC, Receiver Operating Characteristic; AUROC, Area Under the Receiver Operating Characteristic Curve; LASSO-LR, LASSO-Logistic Regression.

**Fig. 5:**
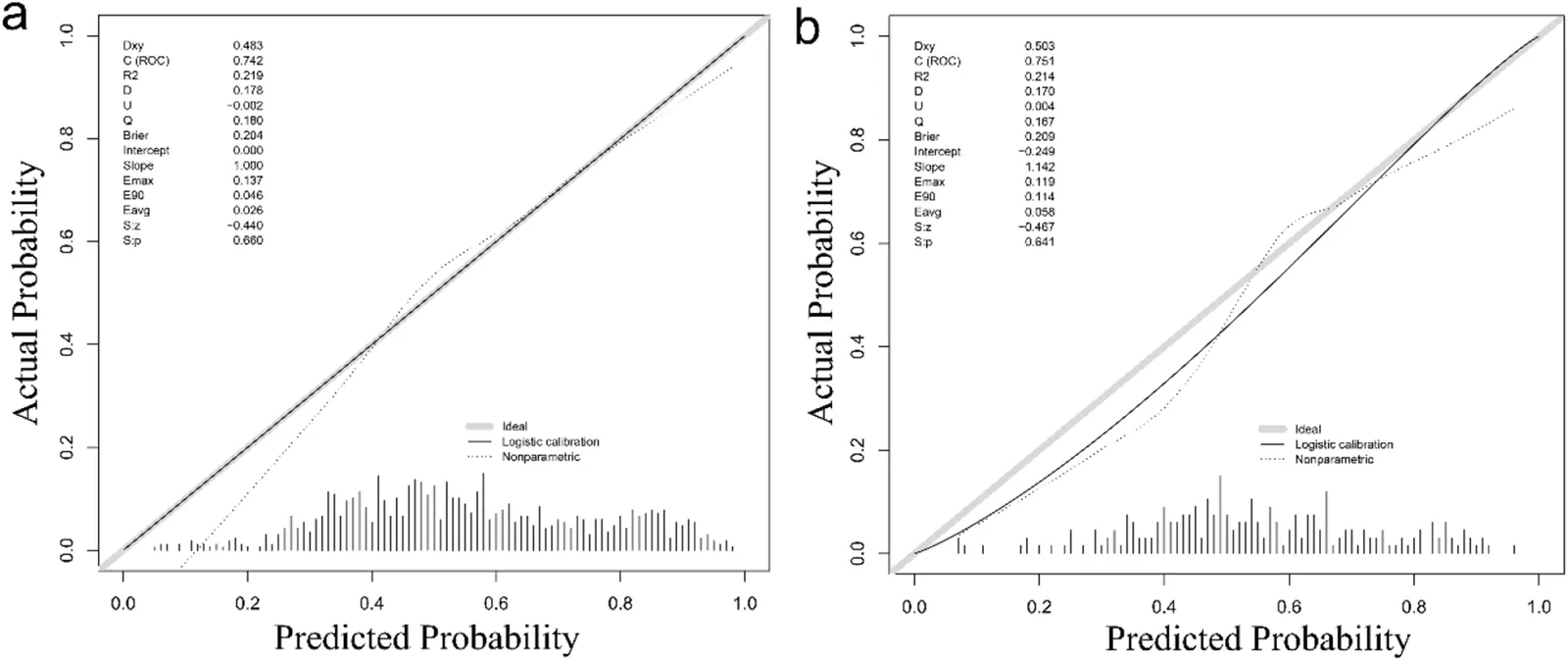
Calibration curves of the lasso-LR model in the training cohort and temporal validation cohort. LASSO-LR, LASSO-Logistic Regression.

**Fig. 6:**
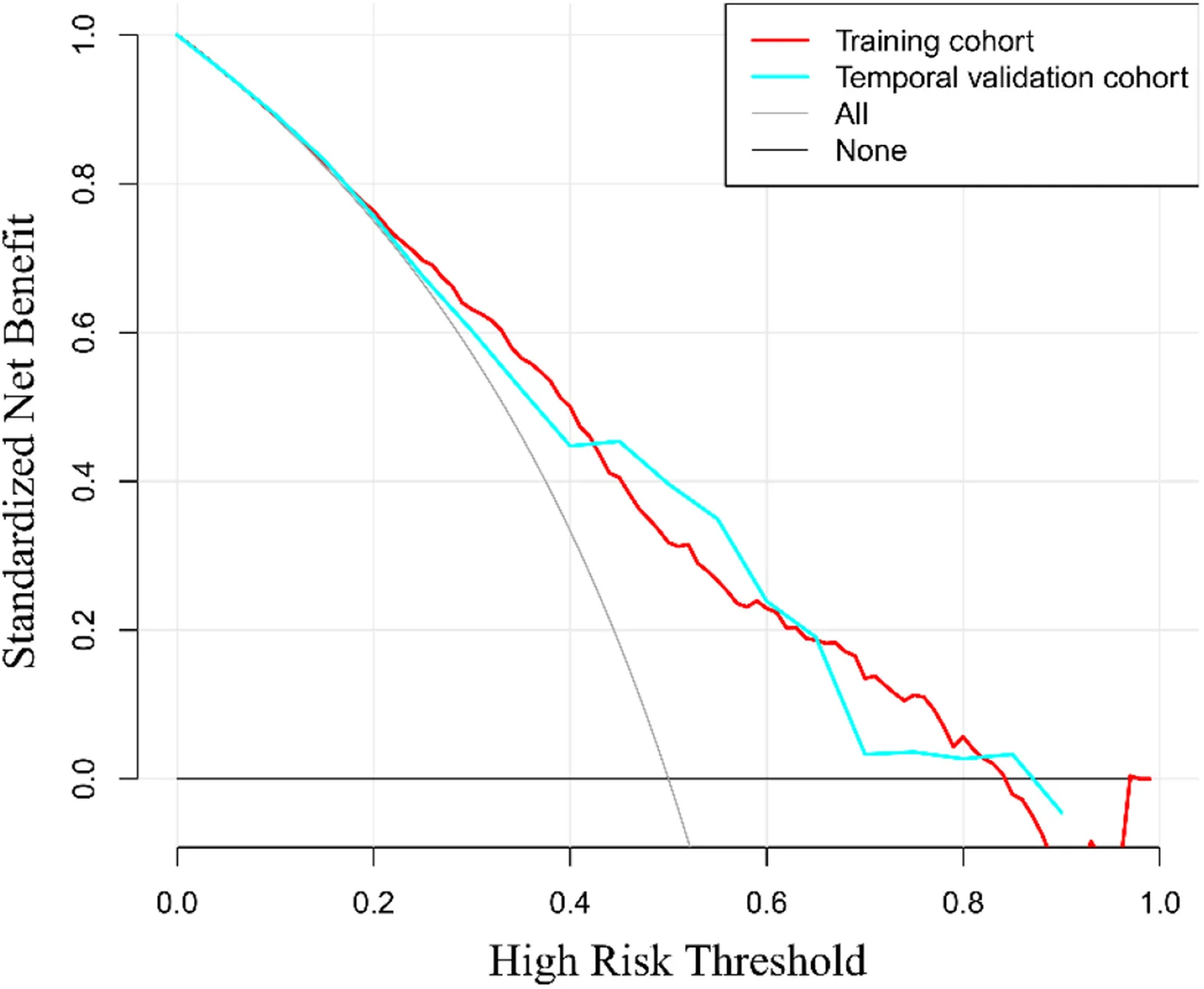
Decision curve analysis of the LASSO-LR model in the training cohort and temporal validation cohort. LASSO-LR, LASSO-Logistic Regression.

In this study, a nomogram tool was utilized to convert key predictors derived from the LASSO-LR model into a mortality risk assessment for patients with CA complicated by AKI. This nomogram assigns specific scores to each predictive variable, sums these scores to obtain a total score, and subsequently translates this total score into a probability of mortality. The model was simplified into a graphical nomogram using R software to facilitate interpretation, as illustrated in Fig. 3. The nomogram demonstrated acceptable discrimination power in the training cohort (AUROC = 0.742, 95% CI: 0.710‒0.774) and the temporal validation cohort (AUROC = 0.751, 95% CI: 0.687‒0.816), as illustrated in Fig. 4. The calibration curve analysis presented in Fig. 5 indicates that the model achieved satisfactory goodness-of-fit across both independent datasets, with Brier scores of 0.204 and 0.209, respectively. These scores indicate a reasonable alignment between predicted and observed outcomes. Given that the binary outcome prevalence in our cohort is approximately 55%, a null model predicting a probability of 0.5 for all patients would result in a Brier score of 0.25. Consequently, the Brier scores of our model signify a marked improvement over the null model, although they also suggest that the probabilistic predictions are not perfectly precise. Furthermore, the DCA curve suggests that the model's prediction for 30-day mortality offers an improved net benefit compared to the “treat all” or “treat none” strategies across a wide range of threshold probabilities. When directly compared with the SOFA score in the training cohort, the model provided a higher net benefit across the majority of clinically relevant threshold probabilities, indicating incremental clinical utility over this existing severity score (Supplementary Fig. 13). This underscores the model's clinical utility in both the training and validation datasets, as depicted in Fig. 6.

To enhance clinical accessibility, we constructed a dynamic online nomogram based on the LASSO-LR model. This interactive tool allows clinicians to input patient-specific variables and obtain an individualized 30-day mortality risk estimate. The dynamic nomogram is available at https://yuanlinx.shinyapps.io/cardiacarrestAKIDynNomapp.

## Discussion

This study developed and validated multiple machine learning models to predict the 30-day mortality rate of patients with CA complicated by AKI. Through analysis of clinical data from the MIMIC-IV database, we determined that the LASSO-LR model demonstrated the most stable performance across cohorts, with superior resistance to overfitting compared to complex models such as KNN and DT. The model’s statistically significant improvement over the APACHE II and SOFA scores was confirmed in the validation cohort. These results suggest modest improvements in prediction accuracy and clinical utility. A sensitivity analysis excluding APACHE II from the LASSO feature pool yielded largely consistent predictors and comparable predictive performance, further supporting the robustness of our findings and suggesting that the inclusion of the composite score did not artificially drive the results. Collectively, these findings offer clinicians a valuable decision support tool that can enhance patient treatment strategies and management.

Through SHAP analysis, seven critical features significantly influencing the 30-day mortality risk for CA patients with AKI were identified, leading to the construction of a nomogram model based on these features. The model showed that temporary pacemaker implantation was associated with a lower SHAP value, whereas oliguria, elevated APACHE II scores, norepinephrine administration, increased anion gap, elevated lactate levels, and heightened RDW were correlated with an increased risk of 30-day mortality. The hierarchy of feature importance was oliguria, RDW, APACHE II score, norepinephrine, anion gap, lactate, and temporary pacemaker implantation. Oliguria was identified as the most critical predictor in the LASSO-LR model, highlighting its significant role in the early diagnosis and prognostic assessment of AKI.^24^ Empirical evidence suggests a strong association between oliguria and elevated mortality rates in patients with AKI.^25^ The manifestation of oliguria indicates not only a reduction in renal filtration capacity but also potentially reflects systemic metabolic imbalances and fluid dysregulation, which are critical factors contributing to the increased mortality risk in post-CA patients.^26^ Recognizing oliguria as a mortality risk predictor highlights the importance of optimizing renal perfusion in the management of these patients.

The APACHE II score, a widely acknowledged severity-of-disease classification system, quantifies dysfunction across multiple organ systems, with elevated scores being strongly correlated with increased mortality rates. Previous research has established that the APACHE II score not only predicts the severity and neurological outcomes in patients with CA,^27^^,^^28^ but also possesses prognostic significance for adverse outcomes in patients with AKI.^29^ This study corroborates its efficacy in prognosticating the outcomes of patients with CA complicated by AKI. Norepinephrine, a potent vasoactive agent, is recommended as a first-line therapeutic option for hemodynamic stabilization in instances of cardiogenic shock or other hypotensive conditions.^30^ The administration of norepinephrine, indicative of hemodynamic instability, is correlated with an elevated mortality risk in this study, aligning with findings from prior research on patients with CA.^31^ Furthermore, a study examining early mortality (within 7-days) in critically ill patients with AKI identified the use of norepinephrine bitartrate as an independent risk factor for mortality.^32^ In patients with CA complicated by AKI, the administration of norepinephrine may indicate severe hemodynamic instability, frequently associated with reduced cardiac output, insufficient tissue perfusion, and multi-organ dysfunction, thereby elevating the risk of mortality. In this study, norepinephrine was treated as a binary variable (use vs. no use) because dosing data in the MIMIC-IV database are not uniformly recorded across patients, making binary categorization more reliable and consistent with prior MIMIC-based studies.^33^^,^^34^

Additionally, the anion gap, lactate levels, and RDW were significant within the model. The anion gap, which reflects acid-base balance, is commonly utilized to evaluate metabolic acidosis. An elevated anion gap is indicative of lactic acidosis, ketoacidosis, or other metabolic abnormalities.^35^ In patients with CA complicated by AKI, an elevated anion gap may indicate lactic acidosis or the accumulation of acidic metabolic products due to renal insufficiency, thereby suggesting an increased risk of mortality.^36^, ^37^, ^38^ Blood lactate levels serve as sensitive indicators of tissue perfusion and oxygenation status, with numerous studies demonstrating that elevated lactate levels are closely associated with adverse outcomes in critically ill patients.^39^ During CA, severe hypoxemia and ischemia-reperfusion injury contribute to elevated lactate levels. The RDW reflects the variability in red blood cell size and has been associated with poor prognosis in patients with CA,^40^ severe AKI,^41^ and heart failure.^42^ Research suggests that elevated RDW is correlated with systemic inflammatory response, oxidative stress, and increased levels of tumor necrosis factor.^43^ In patients with CA complicated by AKI, elevated RDW may indicate systemic inflammation and stress, thereby heightening the risk of mortality. The integrated use of these biomarkers offers clinicians a multidimensional risk assessment, facilitating a more comprehensive understanding of the patient's condition.

The improved discrimination of the LASSO-LR model compared to traditional scoring systems such as APACHE II and SOFA may be attributed to its capacity to incorporate specific, high-impact variables pertinent to this subpopulation. While APACHE II score offers a comprehensive physiological evaluation, our model captures pathophysiological subtleties through variables such as oliguria and RDW, which hold particular significance for patients with CA complicated by AKI. Importantly, the model's Brier scores, ranging from 0.204 to 0.209, demonstrate a marked improvement over the null model benchmark of 0.25. However, these scores also suggest that the probabilistic predictions are not optimally sharp, indicating potential areas for further enhancement. Therefore, individual risk predictions from our model should be interpreted as approximate guides rather than exact probabilities. Small differences in predicted risk (e.g., 68% vs. 72%) are unlikely to be clinically meaningful. We caution against over-interpretation of small differences in total points or predicted risks.

In this study, the implantation of a temporary pacemaker was inversely associated with the 30-day mortality risk. However, this association should be interpreted with caution. Baseline comparisons show that the validation cohort had significantly higher norepinephrine use-a marker of greater severity-yet similar pacemaker use, indicating a complex selection process rather than a straightforward marker of lower severity. Hence, the pacemaker variable likely reflects unmeasured clinical factors and should not be viewed as having a direct protective effect. The variable’s interpretation remains opaque, which limits the clinical interpretability of the nomogram.

The findings of this study may have implications for clinical practice. Firstly, it represents the inaugural integration of advanced machine learning techniques with clinical data to establish a novel method for assessing mortality risk in patients with CA complicated by AKI. This integration aids clinicians in making more precise judgments in complex clinical scenarios. Secondly, the application of SHAP facilitates a deeper understanding of the predictive factors, thereby informing clinical decision-making processes. Furthermore, the implementation of a nomogram enables physicians to rapidly and intuitively evaluate mortality risk, thereby enhancing the efficiency of clinical decision-making.

### Limitations

However, this study is subject to several limitations. Firstly, although a temporal validation strategy was employed, both cohorts were sourced from a single center, which restricts the generalizability of the findings to other populations and settings. Therefore, multi-center external validation is critically needed prior to clinical implementation. Secondly, the study was unable to compare the model with cardiac arrest-specific scores, such as CAHP and OHCA, due to the absence of key variables (e.g., no-flow duration) in the MIMIC-IV database. Thirdly, with an EPV of approximately 72 in the training cohort, the sample size was adequate for stable estimation of the LASSO-logistic regression model; nevertheless, complex machine learning algorithms with numerous hyperparameters may still be prone to overfitting in the absence of very large datasets. Fourthly, associations involving interventions (e.g., temporary pacemaker implantation) are subject to unmeasured selection biases and should not be interpreted as causal. Sensitivity analyses confirmed that the model performance was not driven by this variable, although its inclusion in the nomogram limits straightforward clinical interpretation. Fifth, the study did not account for certain critical but unavailable variables, such as socioeconomic status and long-term health behaviors, which could potentially impact the comprehensiveness of the predictions. Sixth, although the LASSO-selected features were stable across imputed datasets, the model's performance metrics were derived from a single representative dataset rather than pooled across all imputations, which may slightly underestimate prediction interval uncertainty. Future research should employ multicenter and prospective designs to validate and expand upon the findings.

## Conclusions

This study developed and validated machine learning models for predicting 30-day mortality in patients with CA complicated by AKI. The LASSO-LR model demonstrated stable performance with superior resistance to overfitting, offering a modest but statistically significant improvement over traditional clinical scores (APACHE II and SOFA). SHAP analysis and a nomogram enhanced model interpretability, but the clinical meaningfulness of this incremental benefit requires prospective validation before clinical implementation.

## Ethics approval and consent to participate

The database was approved for research use by the Institutional Review Boards of the Massachusetts Institute of Technology and Beth Israel Deaconess Medical Center, and studies using the database were granted a waiver of informed consent. All methods were performed in accordance with the relevant guidelines and regulations.

## Consent to publication

All authors give consent for publication.

## Data availability

The datasets generated and/or analyzed during the current study are available from the corresponding author on reasonable request.

## Authors’ contributions

Meng Yuan: Conceptualization; data curation; formal analysis; writing-original draft.

Lei Zhong: Data curation; methodology; software; validation; visualization; writing-review & editing.

Jie Min: Formal analysis; resources; writing-original draft.

Mingxia Ni: Investigation; writing-original draft.

Yufeng Yao: Conceptualization; project administration; validation; writing-review & editing.

## Funding

This work was supported by the 10.13039/501100013052Medical and Health Research Project of Zhejiang Province (2025KY1546).

## Declaration of competing interest

The authors declare that they have no known competing financial interests or personal relationships that could have appeared to influence the work reported in this paper.

## Appendix Supplementary materials

Image, application 1
