# Supplementary material for: Development and validation of machine learning models to predict 30-day mortality in patients with cardiac arrest complicated by acute kidney injury
Source: Clinics (Sao Paulo). 2026 Sep 8;81:101129. doi: 10.1016/j.clinsp.2026.101129 (PMC13573679; doi:10.1016/j.clinsp.2026.101129)
Supplement: Supplementary file 1 [file mmc1.docx]

**Supplementary Material**

**(Supplementary Figures 1-17 and Supplementary Tables 1-5)**

**Supplementary Figure 1** The dataset before (a) and after (b) multiple imputation.

**Supplementary Figure 2** Clinical features were selected based on LASSO regression with cross-validation. (a) Selection of optimal parameter lambda (cross-validation); (b) Dynamic process diagram of feature selection using LASSO regression. LASSO, least absolute shrinkage and selection operator.

**Supplementary Figure 3** AUROCs of six machine learning models in the temporal validation cohort. AUROC, area under the receiver operating characteristic curve; Lasso-LR, lasso-logistic regression; DT, decision tree; KNN, K-nearest neighbor; LightGBM, light gradient boosting machine; XGBoost, extreme gradient boosting; SVM, support vector machine.

**Supplementary Figure 4** Calibration curves of six machine learning models in the temporal validation cohort. Lasso-LR, lasso-logistic regression; DT, decision tree; KNN, K-nearest neighbor; LightGBM, light gradient boosting machine; XGBoost, extreme gradient boosting; SVM, support vector machine.

**Supplementary Figure 5** Decision curve analysis of six machine learning models in the temporal validation cohort. Lasso-LR, lasso-logistic regression; DT, decision tree; KNN, K-nearest neighbor; LightGBM, light gradient boosting machine; XGBoost, extreme gradient boosting; SVM, support vector machine.

**Supplementary Figure 6** AUROCs of APACHE II sore in the training cohort and the temporal validation cohort. AUROC, area under the receiver operating characteristic curve; APACHE II, Acute Physiology and Chronic Health Evaluation II.

**Supplementary Figure 7** Calibration curves of APACHE II sore in the training cohort and the temporal validation cohort. APACHE II, Acute Physiology and Chronic Health Evaluation II.

**Supplementary Figure 8** Decision curve analysis of APACHE II sore in the training cohort and the temporal validation cohort. APACHE II, Acute Physiology and Chronic Health Evaluation II.

**Supplementary Figure 9** AUROCs of SOFA sore in the training cohort and the temporal validation cohort. AUROC, area under the receiver operating characteristic curve; SOFA, Sequential Organ Failure Assessment.

**Supplementary Figure 10** Calibration curves of SOFA sore in the training cohort and the temporal validation cohort. SOFA, Sequential Organ Failure Assessment.

**Supplementary Figure 11** Decision curve analysis of SOFA sore in the training cohort and the temporal validation cohort. SOFA, Sequential Organ Failure Assessment.

**Supplementary Figure 12** The influence of seven distinct features on the predicted outcomes. The horizontal axis displays the values of continuous variables or the presence/absence of categorical variables, whereas the vertical axis denotes the SHAP values. SHAP, SHapley Additive exPlanations; APACHE II, Acute Physiology and Chronic Health Evaluation II.

**Supplementary Figure 13** Decision curve analysis of the Lasso-LR model and the SOFA sore in the training cohort. SOFA, Sequential Organ Failure Assessment.

**Supplementary Figure 14** LASSO feature selection after TPI exclusion. (a) Selection of optimal parameter lambda (cross-validation); (b) Dynamic process diagram of feature selection using LASSO regression. LASSO, least absolute shrinkage and selection operator; TPI, temporary pacemaker implantation.

**Supplementary Figure 15** ROC curves of six prediction models in the TPI‑excluded validation cohort. ROC, receiver operating characteristic; TPI, temporary pacemaker implantation.

**Supplementary Figure 16** Calibration curves of six prediction models in the TPI‑excluded validation cohort. TPI, temporary pacemaker implantation.

**Supplementary Figure 17** Decision curve analysis for six prediction models in the TPI‑excluded validation cohort. TPI, temporary pacemaker implantation.

| **Supplementary Table 1** Summary of missing data for candidate variables. | | |
| --- | --- | --- |
| **Variables** | **Missing (n)** | **Missing (%)** |
| APACHE II score | 61 | 5.44% |
| Baseline creatinine | 2 | 0.18% |
| Heart rate | 3 | 0.27% |
| MAP | 3 | 0.27% |
| PaO2 | 91 | 8.12% |
| Lactate | 143 | 12.76% |
| Anion gap | 6 | 0.54% |
| WBC count | 4 | 0.36% |
| RBC count | 3 | 0.27% |
| Hemoglobin | 3 | 0.27% |
| Platelet count | 3 | 0.27% |
| RDW | 3 | 0.27% |
| BUN | 2 | 0.18% |
| Creatinine | 3 | 0.27% |
| ALT | 238 | 21.23% |
| AST | 235 | 20.96% |
| Total bilirubin | 246 | 21.95% |
| Prothrombin time | 15 | 1.34% |
| Glucose | 6 | 0.54% |
| Calcium | 41 | 3.66% |
| Sodium | 5 | 0.45% |
| Chloride | 6 | 0.54% |
| Potassium | 5 | 0.45% |
| Magnesium | 25 | 2.23% |
| APACHE II Acute Physiology and Chronic Health Evaluation II, MAP mean arterial pressure, PaO2 partial pressure of arterial oxygen, WBC white blood cell, RBC red blood cell, RDW red cell distribution width, BUN blood urea nitrogen, ALT alanine transaminase, AST aspartate transaminase. | | |

| **Supplementary Table 2** Characteristics of the participants in different groups. | | | | | |
| --- | --- | --- | --- | --- | --- |
| **Characteristic** | **Total**  **(n=1,121)** | **Training cohort**  **(n=900)** | **Temporal validation cohort**  **(n=221)** | **t/Z/χ2** | ***P* value** |
| Age (years) | 66.98 ± 16.91 | 67.76 ± 16.68 | 63.82 ± 17.50 | 3.113 | 0.002 |
| Male, n (%) | 677 (60.39) | 542 (60.22) | 135 (61.09) | 0.055 | 0.814 |
| Marital status |  |  |  | 44.813 | <0.001 |
| Divorced, single, widowed | 450 (40.14) | 381 (42.33) | 69 (31.22) |  |  |
| Married | 450 (40.14) | 377 (41.89) | 73 (33.03) |  |  |
| Other | 221 (19.71) | 142 (15.78) | 79 (35.75) |  |  |
| SOFA score | 9.20 ± 4.39 | 9.14 ± 4.43 | 9.42 ± 4.20 | -0.843 | 0.400 |
| APACHE II score | 26.91 ± 8.34 | 27.20 ± 8.55 | 25.74 ± 7.30 | 2.331 | 0.020 |
| 30-day mortality (%) | 613 (54.68) | 502 (55.78) | 111 (50.23) | 2.207 | 0.137 |
| Baseline creatinine (umol/L) | 71.99 (53.04, 95.83) | 73.67 (39.78, 96.98) | 70.72 (35.36, 93.02) | 1.524 | 0.127 |
| **AKI stages, n (%)** |  |  |  | 6.738 | 0.034 |
| Stage 1 | 187 (16.68) | 141 (15.67) | 46 (20.81) |  |  |
| Stage 2 | 485 (43.26) | 383 (42.56) | 102 (46.15) |  |  |
| Stage 3 | 449 (40.05) | 376 (41.78) | 73 (33.03) |  |  |
| Heart rate (min^−1^) | 91.31 ± 22.68 | 91.16 ± 22.46 | 91.92 ± 23.58 | -0.447 | 0.655 |
| MAP (mmHg) | 85.25 ± 20.72 | 85.14 ± 20.72 | 85.67 ± 20.78 | -0.336 | 0.737 |
| PaO2 (mmHg) | 99.00 (62.00, 227.00) | 112.00 (45.00, 245.00) | 73.00 (31.00, 145.00) | 6.665 | <0.001 |
| Lactate (mmol/L) | 3.00 (1.70, 5.80) | 2.90 (1.20, 5.55) | 3.70 (1.40, 6.30) | -3.012 | 0.003 |
| Anion gap (mmol/L) | 17.95 ± 5.67 | 17.94 ± 5.66 | 18.00 ± 5.73 | -0.130 | 0.896 |
| WBC count (×10^9^/L) | 12.60 (8.70, 17.50) | 12.70 (8.75, 17.45) | 12.40 (8.50, 18.30) | -0.363 | 0.717 |
| RBC count (×10^12^/L) | 3.86 ± 0.92 | 3.86 ± 0.90 | 3.89 ± 0.98 | -0.462 | 0.645 |
| Hemoglobin (g/L) | 115.61 ± 27.53 | 115.73 ± 26.98 | 115.14 ± 29.71 | 0.286 | 0.775 |
| RDW (%) | 15.00 ± 2.26 | 15.05 ± 2.24 | 14.81 ± 2.32 | 1.377 | 0.169 |
| Platelet count (×10^9^/L) | 205.00 (149.00, 272.00) | 208.00 (149.00, 274.50) | 197.00 (148.00, 267.00) | 0.979 | 0.327 |
| BUN (mmol/L) | 7.83 (5.70, 12.46) | 8.19 (5.70, 12.46) | 7.12 (5.34, 11.04) | 3.045 | 0.002 |
| Creatinine (umol/L) | 106.08 (79.56, 159.12) | 106.08 (79.56, 159.12) | 106.08 (79.56, 150.28) | 0.792 | 0.429 |
| ALT (U/L) | 58.00 (27.00, 175.00) | 58.00 (28.00, 176.00) | 59.00 (26.00, 173.00) | 0.071 | 0.943 |
| AST (U/L) | 92.00 (41.50, 258.00) | 92.00 (41.50, 258.00) | 108.00 (42.00, 286.00) | -0.824 | 0.410 |
| Total bilirubin (umol/L) | 10.26 (6.84, 17.10) | 10.26 (6.84, 17.10) | 10.26 (5.13, 17.10) | 0.990 | 0.322 |
| Prothrombin time (s) | 14.20 (12.40, 18.20) | 14.40 (12.60, 18.80) | 13.10 (11.80, 16.60) | 4.169 | < 0.001 |
| Glucose (mmol/L) | 9.05 (6.83, 13.05) | 8.94 (6.89, 13.05) | 9.39 (6.78, 13.05) | -0.468 | 0.640 |
| Calcium (mmol/L) | 2.07 ± 0.31 | 2.07 ± 0.32 | 2.09 ± 0.26 | -1.054 | 0.292 |
| Sodium (mmol/L) | 138.59 ± 5.65 | 138.51 ± 5.70 | 138.91 ± 5.44 | -0.920 | 0.358 |
| Chloride (mmol/L) | 103.61 ± 6.78 | 103.93 ± 6.74 | 102.32 ± 6.79 | 3.169 | 0.002 |
| Potassium (mmol/L) | 4.41 ± 1.01 | 4.39 ± 0.99 | 4.50 ± 1.08 | -1.517 | 0.130 |
| Magnesium (mmol/L) | 0.86 ± 0.21 | 0.86 ± 0.21 | 0.86 ± 0.21 | 0.329 | 0.742 |
| **Comorbidities, n(%)** |  |  |  |  |  |
| Obesity | 330 (29.44) | 257 (28.56) | 73 (33.03) | 1.711 | 0.191 |
| Diabetes | 342 (30.51) | 283 (31.44) | 59 (26.70) | 1.886 | 0.170 |
| Hypertension | 495 (44.16) | 418 (46.44) | 77 (34.84) | 9.687 | 0.002 |
| AMI | 250 (22.30) | 180 (20.00) | 70 (31.67) | 13.955 | <0.001 |
| Atrial fibrillation | 397 (35.41) | 335 (37.22) | 62 (28.05) | 6.520 | 0.011 |
| Cardiogenic shock | 201 (17.93) | 174 (19.33) | 27 (12.22) | 6.106 | 0.013 |
| Ventricular fibrillation | 191 (17.04) | 162 (18.00) | 29 (13.12) | 2.987 | 0.084 |
| COPD | 153 (13.65) | 121 (13.44) | 32 (14.48) | 0.161 | 0.688 |
| Pulmonary embolism | 60 (5.35) | 45 (5.00) | 15 (6.79) | 1.119 | 0.290 |
| Cirrhosis | 58 (5.17) | 48 (5.33) | 10 (4.52) | 0.236 | 0.627 |
| Cerebral infarction | 180 (16.06) | 126 (14.00) | 54 (24.43) | 14.332 | < 0.001 |
| SAH | 40 (3.57) | 27 (3.00) | 13 (5.88) | 4.284 | 0.038 |
| Rheumatic disease | 39 (3.48) | 29 (3.22) | 10 (4.52) | 0.897 | 0.344 |
| Malignancy | 141 (12.58) | 112 (12.44) | 29 (13.12) | 0.074 | 0.785 |
| CKD | 235 (20.96) | 191 (21.22) | 44 (19.91) | 0.185 | 0.668 |
| Oliguria | 207 (18.47) | 176 (19.56) | 31 (14.03) | 3.602 | 0.058 |
| Septic shock | 173 (15.43) | 127 (14.11) | 46 (20.81) | 6.109 | 0.013 |
| Depression | 136 (12.13) | 104 (11.56) | 32 (14.48) | 1.423 | 0.233 |
| **Therapeutic interventions, n(%)** | 0 (0.00) |  | 0 (0.00) |  |  |
| Mechanical ventilation | 1,013 (90.37) | 817 (90.78) | 196 (88.69) | 0.890 | 0.345 |
| IABP | 68 (6.07) | 60 (6.67) | 8 (3.62) | 2.891 | 0.089 |
| Temporary pacemaker implantation | 42 (3.75) | 33 (3.67) | 9 (4.07) | 0.081 | 0.776 |
| Defibrillation | 75 (6.69) | 69 (7.67) | 6 (2.71) | 6.969 | 0.008 |
| Norepinephrine | 700 (62.44) | 542 (60.22) | 158 (71.49) | 9.611 | 0.002 |
| Epinephrine | 253 (22.57) | 186 (20.67) | 67 (30.32) | 9.455 | 0.002 |
| TEE | 426 (38.00) | 347 (38.56) | 79 (35.75) | 0.594 | 0.441 |
| Bronchoscopy | 155 (13.83) | 133 (14.78) | 22 (9.95) | 3.464 | 0.063 |
| RRT | 134 (11.95) | 101 (11.22) | 33 (14.93) | 2.320 | 0.128 |
| SOFA Sequential Organ Failure Assessment, APACHE II Acute Physiology and Chronic Health Evaluation II, AKI acute kidney injury, MAP mean arterial pressure, PaO2 partial pressure of arterial oxygen, WBC white blood cell, RBC red blood cell, RDW red cell distribution width, BUN blood urea nitrogen, ALT alanine transaminase, AST aspartate transaminase, AMI acute myocardial infarction, COPD chronic obstructive pulmonary disease, SAH subarachnoid hemorrhage, CKD chronic kidney disease, IABP intra-aortic balloon pump, TEE transthoracic echocardiography, RRT renal replacement therapy. | | | | | |

| **Supplementary Table 3** Comparison of predictive performance between the Lasso-LR model and other models in the training cohort and the temporal validation cohort. | | | | |
| --- | --- | --- | --- | --- |
| **Models** | **AUROC (95% CI)** | **Difference in AUROC (95% CI)** | **Z value** | **P value** |
| **Training cohort** |  |  |  |  |
| Lasso-LR (reference) | 0.742 (0.710-0.774) | - | - | - |
| APACHE II sore | 0.651 (0.615-0.687) | 0.091 (0.061-0.121) | 5.982 | <0.001 |
| SOFA sore | 0.623 (0.587-0.660) | 0.119 (0.089-0.149) | 7.713 | <0.001 |
| Decision tree | 0.828 (0.802–0.854) | -0.086 (-0.114 - -0.058) | -6.035 | <0.001 |
| XGBoost | 0.742 (0.710-0.774) | 0.000 (-0.017 - 0.017) | -0.007 | 0.994 |
| LightGBM | 0.756 (0.725-0.787) | -0.014 (-0.031 - 0.003) | -1.659 | 0.097 |
| Support vector machine | 0.737 (0.705-0.770) | 0.004 (-0.007 - 0.016) | 0.735 | 0.463 |
| K-nearest neighbor | 0.889 (0.869-0.909) | -0.147 (-0.172 - -0.123) | -11.764 | <0.001 |
| **Temporal validation cohort** |  |  |  |  |
| Lasso-LR (reference) | 0.751 (0.687-0.816) | - | - | - |
| APACHE II sore | 0.583 (0.508-0.658) | 0.168 (0.102-0.235) | -4.968 | <0.001 |
| SOFA sore | 0.660 (0.589-0.731) | 0.091 (0.023-0.159) | -2.628 | 0.009 |
| Decision tree | 0.663 (0.592-0.734) | 0.088 (0.019-0.158) | 2.496 | 0.013 |
| XGBoost | 0.724 (0.657-0.791) | 0.027 (-0.013-0.067) | 1.333 | 0.183 |
| LightGBM | 0.731 (0.665-0.797) | 0.020 (-0.018-0.059) | 1.042 | 0.298 |
| Support vector machine | 0.723 (0.656-0.790) | 0.028 (0.003-0.053) | 2.221 | 0.026 |
| K-nearest neighbor | 0.667 (0.596-0.738) | 0.084 (0.029-0.139) | 2.985 | 0.003 |
| AUROC area under the receiver operating characteristic curve, CI confidence interval, XGBoost extreme gradient boosting, LightGBM light gradient boosting machine, Lasso-LR least absolute shrinkage and selection operator-logistic regression; APACHE II Acute Physiology and Chronic Health Evaluation II; SOFA sequential organ failure assessment. | | | | |

| **Supplementary Table 4** Performance comparison of predictive models for 30-day mortality in the training cohort and the temporal validation cohort after exclusion of TPI. | | | | | |
| --- | --- | --- | --- | --- | --- |
| **Models** | **AUROC (95%CI)** | **Sensitivity** | **Specificity** | **Accuracy** | **Kappa** |
| **Training cohort** |  |  |  |  |  |
| Decision tree | 0.735 (0.703-0.767) | 0.776 | 0.593 | 0.698 | 0.374 |
| XGBoost | 0.752 (0.720-0.785) | 0.649 | 0.728 | 0.683 | 0.368 |
| Support vector machine | 0.734 (0.700-0.767) | 0.750 | 0.596 | 0.684 | 0.349 |
| LightGBM | 0.750 (0.718-0.782) | 0.762 | 0.604 | 0.694 | 0.370 |
| K‑nearest neighbor | 0.888 (0.867-0.908) | 0.837 | 0.763 | 0.805 | 0.601 |
| Lasso‑LR | 0.730 (0.697-0.764) | 0.752 | 0.574 | 0.676 | 0.330 |
| **Temporal validation cohort** |  |  |  |  |  |
| Decision tree | 0.614 (0.542-0.686) | 0.673 | 0.451 | 0.566 | 0.125 |
| XGBoost | 0.726 (0.657-0.794) | 0.627 | 0.735 | 0.679 | 0.361 |
| Support vector machine | 0.720 (0.651-0.789) | 0.755 | 0.569 | 0.665 | 0.325 |
| LightGBM | 0.727 (0.658-0.795) | 0.745 | 0.618 | 0.684 | 0.365 |
| K‑nearest neighbor | 0.658 (0.585-0.732) | 0.691 | 0.578 | 0.637 | 0.270 |
| Lasso‑LR | 0.738 (0.671-0.806) | 0.782 | 0.539 | 0.665 | 0.324 |
| TPI, temporary pacemaker implantation, AUROC area under the receiver operating characteristic curve, CI confidence interval, XGBoost extreme gradient boosting, LightGBM light gradient boosting machine, Lasso-LR least absolute shrinkage and selection operator-logistic regression. | | | | | |

| **Supplementary Table 5** Performance comparison of predictive models for 30-day mortality in the training cohort and the temporal validation cohort after recoding temporary pacemaker implantation as “not present” in all patients. | | | | | |
| --- | --- | --- | --- | --- | --- |
| **Models** | **AUROC (95%CI)** | **Sensitivity (%)** | **Specificity (%)** | **Accuracy (%)** | **Kappa** |
| **Training cohort** |  |  |  |  |  |
| Decision tree | 0.831 (0.806-0.857) | 0.693 | 0.791 | 0.737 | 0.476 |
| XGBoost | 0.742 (0.710-0.774) | 0.745 | 0.613 | 0.687 | 0.361 |
| Support vector machine | 0.737 (0.705-0.770) | 0.757 | 0.608 | 0.691 | 0.368 |
| LightGBM | 0.756 (0.725-0.787) | 0.767 | 0.616 | 0.700 | 0.386 |
| K-nearest neighbor | 0.889 (0.869-0.909) | 0.803 | 0.804 | 0.803 | 0.604 |
| Lasso-LR | 0.734 (0.701-0.766) | 0.582 | 0.749 | 0.656 | 0.321 |
| **Temporal validation cohort** |  |  |  |  |  |
| Decision tree | 0.668 (0.597-0.739) | 0.495 | 0.755 | 0.624 | 0.250 |
| XGBoost | 0.724 (0.657-0.791) | 0.739 | 0.627 | 0.683 | 0.366 |
| Support vector machine | 0.723 (0.656-0.790) | 0.757 | 0.582 | 0.670 | 0.339 |
| LightGBM | 0.731 (0.665-0.797) | 0.730 | 0.618 | 0.674 | 0.348 |
| K-nearest neighbor | 0.667 (0.596-0.738) | 0.631 | 0.618 | 0.624 | 0.249 |
| Lasso-LR | 0.741 (0.676-0.807) | 0.622 | 0.745 | 0.683 | 0.367 |
| TPI, temporary pacemaker implantation, AUROC area under the receiver operating characteristic curve, CI confidence interval, XGBoost extreme gradient boosting, LightGBM light gradient boosting machine, Lasso-LR least absolute shrinkage and selection operator-logistic regression. | | | | | |
